# Perceived impact of the COVID-19 pandemic and government restrictions on the lives of young adults living with HIV in Kisumu, Kenya

**DOI:** 10.1371/journal.pgph.0004064

**Published:** 2024-12-13

**Authors:** Jennifer M. Zech, Allison Zerbe, Michael Mangold, Steve Akoth, Redempta David, Judith Odondi, Doris Naitore, Kelvin Ndede, Allison Hsu, Mark Hawken, Tiffany G. Harris, Elaine J. Abrams

**Affiliations:** 1 ICAP at Columbia University, Mailman School of Public Health, New York, New York, United States of America; 2 Columbia University Irving Medical Center, New York, New York, United States of America; 3 ICAP at Columbia University, Nairobi, Kenya; 4 Department of Epidemiology, Columbia University, New York, New York, United States of America; 5 Department of Pediatrics, Vagelos College of Physicians and Surgeons, Columbia University, New York, New York, United States of America; University of Waterloo School of Public Health and Health Systems, CANADA

## Abstract

Young adults with HIV (YAHIV) may be particularly vulnerable to the impact of the COVID-19 pandemic. In this context, associated mitigation measures among YAHIV can adversely impact fragile social and economic systems. We examined the impact of the pandemic and related government-mandated restrictions among YAHIV in Kisumu, Kenya. Between April-May 2021, a cross-sectional survey was conducted among a convenience sample of YAHIV 18–25 years receiving HIV care in Kisumu, Kenya. The information collected included demographics, COVID-19 knowledge, protective measures, and the impact of the pandemic and related restrictions on their daily lives and well-being since the start of the pandemic (i.e., curfews, lockdowns, school/workplace closures). Responses were analyzed using descriptive statistics. Of 275 YAHIV: median age 22 years (IQR: 19–24 years); 178 (65%) female; 222 (81%) completed some secondary education or higher; 108 (39%) lived in an informal housing area. Awareness of COVID-19 was high (99%), mean knowledge score was 4.32 (SD: 0.93; range 1–5) and most reported taking protective measures. Overall, 193 (70%) reported they were affected by COVID-19 and associated restrictions. Almost half (49%) reported changes in a living situation; 24% living with different people, 11% had moved/relocated, and 5% were newly living on the street. Additionally, respondents reported increased verbal arguments (30%) and physical conflict (16%) at home with 8% reporting someone having used/threatened them with a weapon, 12% experiencing physical abuse, 7% being touched in a sexual way without permission, and 5% had forced sex. Impacts of the pandemic and related restrictions were felt across various aspects of YAHIV’s lives, including disrupted living situations and increased exposure to verbal and physical conflict, including sexual violence. Interventions are needed to address the impact and potential negative long-term effects of the pandemic on YAHIV health and well-being.

## Introduction

Young people have been impacted by the global COVID-19 pandemic and associated government restrictions in many ways including increased mental health issues, social and education disruptions, and economic vulnerability [1–3]. Studies conducted globally, including in Africa, show increased experiences of stress, anxiety, and depression among young people due to COVID-19 including feelings of loneliness, fear, and hopelessness [4–9]. Food shortages and lack of access to basic needs are among the economic impacts experienced by young people [10,11]. Disruptions in education were widespread with high rates of full day school closures, an average of 101 days from March 2020 to February 2021 in countries in the Eastern and Southern Africa region [12]. Reaching students by digital or remote learning programs was a challenge and students also struggled adapting to the virtual learning environment [9,13,14]. Globally, data indicates that the impacts of COVID-19 have also been experienced differently between males and females, with young women often experiencing more negative effects [15–17].

People living with HIV were also specifically impacted by COVID-19 as this group faced a higher risk of experiencing severe outcomes from COVID-19 [18,19]. Young people living with HIV may be particularly vulnerable to the negative effects of COVID-19 and associated mitigation measures as many face challenges of poverty, stigma, and living with a chronic disease requiring adherence to daily antiretroviral therapy (ART) [20–23]. Furthermore, retaining adolescents and youth in chronic care is often challenging [24–27], and exacerbated by humanitarian emergencies, large-scale disasters, and infectious outbreaks [28–31].

In response to COVID-19, national governments implemented a variety of public health measures to mitigate impact among communities and the health system, including travel restrictions, nationwide lockdowns, and isolation and quarantine measures [32]. The first case of COVID-19 was confirmed in Kenya on 12 March 2020 [33]. The response to COVID-19 by the Kenyan Government was swift. By March 20th, it had established rigorous guidance restricting international and national travel, and implementing social distancing, and curfews for citizens and businesses [34]. Additionally, the government managed a national health education media campaign to educate Kenyans on prevention measures, including behavioral messaging that encouraged Kenyans to stay home and avoid public transportation. To maintain HIV service delivery efforts, the Ministry of Health (MOH) expanded policies to maintain comprehensive care for clients and protect the health care system by reducing facility visit frequency including expansion of multi-month dispensing (MMD) polices to provide 3-months of ART regardless of age and viral load status [35]. Subsequently, Kenya experienced three additional COVID-19 waves in July/August 2020 (7-day average of daily cases at peak: 660 cases), October/November 2020 (7-day average of daily cases at peak: 1,108 cases) and March/April 2021 (7-day average of daily cases at peak: 1,354 cases) [36,37]. During this timeframe, the government restrictions varied as some were lifted or reduced. In response to the third COVID-19 wave in March/April 2021, the cessation of movement into and out of disease-infected counties (Nairobi, Kajiado, Machakos, Kiambu, and Nakuru) was specifically implemented, then lifted on May 1, 2021. All forms of public gathering, especially political rallies, were banned on March 12, 2021. Kenya started administering COVID-19 vaccinations on March 5, 2021, to priority populations, including frontline healthcare workers, uniformed officers, teaching staff, religious leaders, and citizens above 58 years [38].

Several studies have documented the impact of the COVID-19 pandemic on the economic, social, and mental health of young people in Kenya [39,40]. In general, participants reported significant disruptions to their health, well-being, and economic security, including the inability to meet basic economic needs, decreased income, and a lack of access to sexual and reproductive health services. Despite the high burden of HIV among young people in Kenya, little is known about their experiences throughout the COVID-19 pandemic [41,42]. In 2021, an estimated 1.4 million adolescents and adults aged 15 years and older were living with HIV in Kenya [43]. In 2018, the Kenya Population-based HIV Impact Assessment (KENPHIA) reported a 1.4% HIV prevalence among individuals ages 15–24 years (females: 2.2%, males: 0.6%) [44]. Additionally, about 2 in every 5 adult new HIV infections occurred among youth 15–24 years (40%) in 2017 [45]. In Kenya, there are HIV clinics that provide youth-friendly services [46] and other programs focused on providing support to young people living with HIV [47]. Youth-friendly services offer a strategic approach to support ART adherence and viral suppression among young people living with HIV. These services include dedicated healthcare settings for youth, peer support groups, caregiver engagement, trained healthcare providers, and counseling tailored to young people, all aimed at improving their engagement and health outcomes [48,49].

We report findings from a survey of young adults living with HIV (YAHIV) aged 18–25 years enrolled in HIV care at a large hospital in Kisumu, Kenya. The study aimed to assess YAHIV experiences related to COVID-19 and government restrictions; perceived impact of COVID-19 on their health and well-being; knowledge of, attitudes towards, and practices regarding COVID-19. Our findings provide a more robust understanding of how this more vulnerable population experienced the pandemic and perceived related government-mandated restrictions to impact their lives.

## Materials and methods

### Participants and procedures

The study was conducted in April-May 2021 at the Jaramogi Oginga Odinga Teaching and Referral Hospital (JOOTRH) in Kisumu, Kenya. At the time, ICAP at Columbia University was the President’s Emergency Program for AIDS Relief (PEPFAR) implementing partner supporting HIV services at JOOTRH including the Adolescent Patient Support Center (APSC) where participants were recruited. All YAHIV 18–25 years of age who were currently engaged in HIV care at APSC were considered potentially eligible to participate.

Clinic staff provided existing peer educators working at the APSC, who were also YAHIV, with a list of current patients who fit the eligibility criteria. A total of 12 peer educators were trained on study-specific procedures and good clinical practice, emphasizing procedures to avoid coercion among the study population ensuring participants clearly understood their right to decline and received unbiased information about the study’s risks and benefits, allowing for a voluntary decision. The peer educators conducted phone-based recruitment using approved scripts and invited those who were interested to come to the APSC. Upon arrival at APSC, clinic staff confirmed YAHIV eligibility and referred them to the peer educators who provided additional information about study procedures. Study visits were staggered to avoid crowding and personal protective equipment (PPE) was provided to all staff and YAHIV participants. Study procedures were conducted outdoors with the use of privacy barriers.

Prior to initiation of the survey, interested YAHIV read through the informed consent form on the tablet and were asked to indicate their consent by clicking the “I agree” or “I decline” options on the screen. During the recruitment and consenting process, it was made clear to potential participants that their decision to participate in the study would have no impact on the care they received at the APSC. Once consent was obtained, peer educators invited participants to a private, socially distanced area and provided instructions for self-administration of the tablet-based survey. Peer educators were available to participants during survey administration to answer questions or troubleshoot any issues. For those participants who preferred not to self-administer the survey, peer educators administered the survey directly. All study procedures were conducted in English. After completion, participants were compensated with the equivalent of $10 USD for their time.

### Survey measure

The quantitative survey consisted of 107 close-ended questions adapted from multiple survey tools including the KENPHIA household questionnaire [44], World Health Organization (WHO) tool for behavioral insights on COVID-19 [50], and the 2020 Household Pulse Survey [51]. Survey questions focused on individual-level factors including demographic characteristics, household information, educational status, as well as inter-personal factors including relationship status. COVID-19 knowledge, attitudes, perceptions, adherence to precautions, and the perceived impact of COVID-19 and related government restrictions were assessed in the survey. More specifically, questions on the domains of housing, education, employment, finances, food access, access to HIV care, safety/violence, HIV stigma, and mental health during COVID-19 were presented in the survey. Mental health screening questions included validated standards from the generalized anxiety disorder 2-item (GAD-2) [52] and the patient health questionnaire 2-item (PHQ-2) [53]. The survey tool was pilot tested at APSC among the study population and updated based on feedback.

### Data analysis

We present descriptive data on the characteristics of YAHIV 18–25 years of age. We also examined participant characteristics and their COVID-19 knowledge, attitudes, and practices. Descriptive analyses were conducted using SAS (Version 9.4, SAS Institute Inc., Cary, NC, USA). P-values estimating the extent to which random variability can explain observed differences by sex were estimated using Chi-square or Fisher’s Exact statistics for categorical variables, and t-statistics for continuous variables. We also examined characteristics according to sex; p-values <0.05 are noted in the tables and footnotes for categorical variables.

We measured knowledge about COVID-19 using ‘true’/’false’/‘prefer not to answer’ responses to statements about COVID-19 transmission and prevention using questions adapted from previous COVID-19 surveys [40]. For the analysis, we dichotomized responses into incorrect/‘prefer not to answer’ and correct for each question. For mental health screening, GAD-2 and PHQ-2 scores were totaled and analyzed using a total score of 3 or greater as showing signs of possible generalized anxiety disorder (GAD-2) [52] and possible depressive disorder (PHQ-2) [53] to be further evaluated.

### Ethical approvals

The study protocol was approved by the Institutional Review Boards/Ethics Committees at the Columbia University Irving Medical Center (AAAT2632) and the Maseno University Ethics and Research Committee (MUERC/00886/20).

## Results

### Characteristics of study participants

An estimated 407 YAHIV aged 18–25 active on ART were registered at the health facility where the survey was implemented. A total of 364 YAHIV were contacted by peer educators and 43 did not have current contact information. Of those contacted, 292 were eligible for the study and 275 enrolled. A total of 275 YAHIV completed the survey from April-May 2021. Median age was 22 years (interquartile range [IQR] = 19–24 years) and 178 (65%) were female. Most (82%) had completed secondary education or higher. At the time of the survey, 40% were living in an informal settlement (slum) in Kisumu, of which nearly half (48%) lived in the Manyatta slum. An additional 22% lived in uptown Kisumu in permanent housing while 38% reported living in villages outside of Kisumu City. Two-thirds (66%) had a boyfriend or girlfriend and over one-third (39%) of YAHIV had children with 14% having more than one child. Female participants were significantly more likely to report having a boyfriend/girlfriend as well as having at least one living child (Table 1).

**Table 1:** Demographic characteristics of young adults with HIV (18–25 years) completing the cross-sectional survey in Kisumu, Kenya, April-May 2021 (N = 275).

	Total		Male		Female	
	N	%	N	%	N	%
	275	100%	97	35%	178	65%
Age, median (IQR)	22 (19–24)		20 (19–23)		22.5 (20–24)	
Highest level of education (N = 270)	270	98%	95	98%	175	98%
*Nursery/Preschool—Primary Class 4*	3	1%	0	0%	3	2%
*Primary Class 5–8*	45	17%	10	11%	35	20%
*Secondary Form 1–4*	135	50%	53	56%	82	47%
*Technical School*	16	6%	5	5%	11	6%
*University*	33	12%	14	15%	19	11%
*College*	38	14%	13	14%	25	14%
Residential area (currently living) (N = 272)	272	99%	96	99%	173	97%
*Village outside Kisumu city*	104	38%	38	40%	66	38%
*Up Town (permanent housing)*	60	22%	27	28%	33	19%
*Informal settlement (slum)*	108	40%	31	32%	77	44%
Has boyfriend / girlfriend* (N = 259)	259	94%	90	93%	169	95%
*Yes*	170	66%	51	57%	119	70%
*No*	89	34%	39	43%	50	30%
Number of living children*						
*0*	168	61%	87	90%	81	46%
*1*	70	25%	6	6%	64	36%
*2*	29	11%	4	4%	25	14%
*3*	5	2%	0	0%	5	3%
*4 or more*	3	1%	0	0%	3	2%
Total number of people in household (including self) (N = 274)	274	99%	96	99%	178	100%
*1 to 3*	68	25%	26	27%	42	24%
*4 to 6*	128	47%	49	51%	79	44%
*7 or more*	78	28%	21	22%	57	32%
Number of rooms in household* (N = 260)	260	95%	91	94%	169	95%
*1*	124	48%	34	37%	90	54%
*2*	84	32%	31	34%	53	31%
*3*	36	14%	17	19%	19	11%
*4 or more*	16	68%	9	10%	7	4%

* = p-value < 0.05.

### COVID-19 information, knowledge and prevention practices

Almost all (99%) participants had heard of COVID-19 with most getting COVID-19-related information across multiple sources, including television, radio, social media, and/or conversations with family/friends and health workers. Despite high levels of awareness and multiple information sources, about one-third (31%) of participants reported not currently having enough COVID-19 information and an additional 6% reported being unsure if they had sufficient information. Over half (56%) of the YAHIV (N = 263) trusted the COVID-19 information from the MOH “fully” (32%) or “a lot” (24%). Of the remaining participants (N = 117), 20% were neutral and 19% trusted the information “a little” and 6% did not trust the information. The overall mean COVID-19 knowledge score was 4.32 (SD: 0.93; range 1–5), suggesting an overall 86% correct rate on this knowledge test. More than half (56%) of the participants answered all five questions correctly. Most participants reported frequent handwashing and/or use of hand sanitizer (91%) and wearing face masks (85%) as COVID-19 prevention practices. In general, YAHIV participants reported avoiding large groups/gatherings (77%). However, over half (55%) also reported being in a crowded place within the last 30 days before the survey. Females were more likely than males to express concern about contracting COVID-19 and more likely to implement certain prevention practices.

### Overall perceived impacts of COVID-19 and government restrictions

When asked how COVID-19 and the government restrictions affected them, 30% of YAHIV stated the restrictions did not affect them. However, 70% felt that the restrictions had multiple impacts, mainly on their daily routine (38%) and views on travel and immigration (22%), and relationships (14%), including general positive effects (13%). Perceived impacts on specific aspects of YAHIV lives are described below.

### Perceived impact on education, work, finances, and food

Of the 167 (61%) YAHIV who reported being in school before COVID-19, 29% stopped attending school entirely, 45% switched to remote learning, and 26% reported no change. In addition, YAHIV reported that they, themselves, as well as individuals who support them financially were working less (63%) and earning less income (73%) than before COVID-19. Changes in finances resulted in YAHIV and their households reducing spending, mostly on transportation (44%) and food (42%). Females were more likely than males to report reductions in spending on food. Almost one-third of participants reported that they often (7%) or sometimes (29%) did not have enough food to eat before the pandemic whereas, almost half of the YAHIV reported not having enough to eat often (8%) or sometimes (35%) in the last seven days. When comparing the change in food eaten at the household level from before and during COVID-19, over half (64%) reported no change while the remaining saw a negative (27%) or positive (10%) change. Furthermore, since the start of COVID-19 and associated restrictions, 7% of surveyed YAHIV reported that someone in their household had sex for money, good, gifts, rent, or other items and 9% reported that they had sex in exchange for money and goods. Young women were more likely to report having sex in exchange for money, food, gifts, rent, or other items since COVID-19 and the MOH restrictions began (Table 2).

**Table 2:** Perceived educational and economic impacts of COVID-19 on YAHIV in Kisumu, Kenya, April-May 2021 (N = 275).

	Total		Male		Female			
	N	%	N	%	N			%
	275	100%	97	35%	178			65%
**Education**								
Changes in schooling (N = 167)	167	61%	68	70%		99	56%	
Stopped attending school entirely	49	29%	17	25%		32	32%	
Switched to remote learning	75	45%	34	50%		41	41%	
No change	43	26%	17	25%		26	26%	
**Economic**								
Items YAHIV or family had to cut back spending on								
*Transportation*	121	44%	42	43%		79	44%	
Food*	115	42%	32	33%		83	47%	
Hair dressing salon*	71	26%	10	10%		61	34%	
*Airtime*	64	23%	19	20%		45	25%	
*Hygienic products or toiletries*	45	16%	14	14%		31	17%	
Feminine care products*	41	15%	6	6%		35	20%	
*Alcohol*	9	3%	2	2%		7	4%	
*Cigarettes*	6	2%	3	3%		3	2%	
One statement that best describes the food eaten in your household before the COVID-19 pandemic								
*Enough of the kinds of food (I/we) wanted to eat*	97	35%	40	41%		57	32%	
*Enough*, *but not always the kinds of food (I/we) wanted to eat*	81	29%	32	33%		49	28%	
*Sometimes not enough to eat*	79	29%	20	21%		59	33%	
*Often not enough to eat*	18	7%	5	5%		13	7%	
One statement that best describes the food eaten in your household in last 7 days								
*Enough of the kinds of food (I/we) wanted to eat*	54	20%	26	27%		28	16%	
*Enough*, *but not always the kinds of food (I/we) wanted to eat*	102	37%	36	37%		66	37%	
*Sometimes not enough to eat*	96	35%	26	27%		70	39%	
*Often not enough to eat*	23	8%	9	9%		14	8%	
Change in food eaten in your household (before COVID-19 pandemic vs. in last 7 day)								
*Positive change*	27	10%	7	7%		20	11%	
*Negative change*	73	27%	25	26%		48	27%	
*No change*	175	64%	65	67%		110	62%	
Confidence that your household will be able to afford the kinds of food you need								
*Not at all confident*	115	42%	32	33%		83	47%	
*Somewhat confident*	42	15%	15	15%		27	15%	
*Moderately confident*	81	29%	34	35%		47	26%	
*Very confident*	37	13%	16	16%		21	12%	
Anyone in household (not including the YAHIV) exchanged sex for money, food, gifts, rent or other items since COVID-19 and the Government restrictions began (N = 252)	20	7%	4	4%		16	9%	
YAHIV self-reported having sex for money, food, gifts, rent or other items since COVID-19 and the MOH restrictions began* (N = 271)	25	9%	4	4%		21	12%	

* = p-value < 0.05.

### Perceived impact on healthcare access and outcomes

In the past month, one quarter (25%) of YAHIV experienced a delay in receiving healthcare and 24% did not receive the medical care they needed for something other than COVID-19. Most (81%) YAHIV reported that they had not missed any scheduled HIV appointments since the start of the restrictions. Of the 15% who missed an appointment, the most common reasons included: lack of money or insurance (44%), inconvenience- location/hours (37%), canceled due to COVID-19 (17%), or they forgot (10%). For 26% of YAHIV, COVID-19 and government restrictions made it more difficult to access ART medications and 10% reported missing a dose since the government restrictions started.

Almost one-fourth (23%) of the YAHIV screened positive on GAD-2 for signs of possible generalized anxiety disorder (score ≥3). Similarly, 25% of YAHIV scored between 3–6 on PHQ-2, suggesting evidence of possible depressive disorder to be further evaluated. There were no statistically significant gender differences found among healthcare and mental health impact variables (Table 3).

**Table 3:** Perceived healthcare and mental health impacts of COVID-19 on YAHIV in Kisumu, Kenya, April-May 2021 (N = 275).

	Total		Male		Female	
	N	%	N	%	N	%
	275	100%	97	35%	178	65%
**Healthcare**						
At any time in the past month, experienced delay in getting medical care because of COVID-19	68	25%	22	23%	46	26%
At any time in the past month, need medical care for something other than COVID-19, but did not get it because of COVID-19	66	24%	20	21%	46	26%
Missed any scheduled appointments with at the HIV clinic since the lockdown						
*Yes*, *missed a visit*	41	15%	14	14%	27	15%
*No*, *did not miss a visit*	224	81%	78	80%	146	82%
*Did not have a visit scheduled to miss*	5	2%	2	2%	3	2%
*Do not know if visit missed*	5	2%	3	3%	2	1%
COVID-19 and restrictions make it more difficult to get ARVs/ART (N = 256)	72	28%	26	27%	46	26%
COVID-19 and restrictions cause to miss taking doses of ARVs/ART at any time, or stop all together (N = 258)						
*I have missed doses*	26	10%	14	15%	12	7%
*I stopped taking my ARVs*	3	1%	1	1%	2	1%
*I have not missed any doses*	229	89%	78	84%	151	92%
**Mental Health**						
**GAD-2 Scores (N = 262)**						
*0–2*	200	76%	74	80%	126	73%
*3–6*	62	24%	18	20%	47	27%
**PHQ-2 Scores (N = 263)**						
*0–2*	195	74%	71	77%	124	73%
*3–6*	68	26%	21	23%	47	27%

### Impact on housing, neighborhood safety and violence

**Fig 1:**
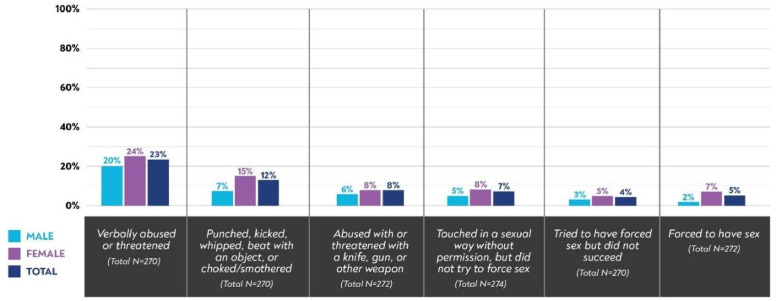
Self-reported experiences of violence since the start of COVID-19 among YAHIV in Kisumu, Kenya, April-May 2021 (N = 275).

Almost half of YAHIV (49%) reported changes in living situation (self or someone in their home), including 24% living with different people, 24% moved, and 10% newly living on the street since the start of COVID-19. Additionally, YAHIV reported increased experiences of verbal arguments in their household toward themselves (14%) and others (17%) as well as an increase in physical conflict toward themselves (6%) and others (10%). Over half of YAHIV reported that their neighborhood was safer (27%) or as safe (33%) since the start of COVID while 40% reported decreased neighborhood safety. Many reported experiences of verbal abuse or threats (23% total: 24% of all females, 20% of all males), or having been punched, kicked, whipped, beaten with an object or choked/smothered (12% total: 15% of all females, 7% of all males), or threatened with a knife, gun or other weapon (8% total: 8% of all females, 6% of all males) (Fig 1). Additionally, some YAHIV reported sexual violence including: someone touched them in a sexual way without permission, but did not try to force sex (7% total: 8% of all females, 5% of all males), someone tried to make them have sex against their will but did not succeed (4% total: 5% of all females, 3% of all males) or someone forced them to have sex (5% total: 7% of all females, 2% of all males). Higher rates of violence were observed among females across all categories; however, none of the gender differences were statistically significant.

## Discussion

The COVID-19 pandemic has been well documented to have broadly disrupted the social fabric, as well as economic, health, and healthcare infrastructure globally [54–57]. Furthermore, adverse consequences of the pandemic and the related mitigation efforts are likely to be most pronounced in low-income countries where the population is already disadvantaged and at risk for poor outcomes. The findings reported here contribute to the emerging body of knowledge around the perceived impacts of COVID-19 in a particularly vulnerable group residing in a low-income country [41,42], namely YAHIV in Kenya. This group is at heightened risk as they are on the cusp of independent adulthood with a chronic, stigmatized disease and a host of existing social and economic vulnerabilities. Overall, we found that YAHIV in Kenya had high rates of COVID-19 knowledge, were aware of the risk of getting COVID-19, and understood practices to minimize this risk but did not always adhere to these practices. YAHIV reported that COVID-19 impacted their economic, mental, and physical well-being and caused specific disruptions in their home lives with reported increases in verbal and physical conflict. While our data did not indicate many significant differences between male and female young adults on perceived impacts of COVID-19, the differences we did see align with other reports [15–17]. By investigating the impact of the pandemic on YAHIV in Kenya, results from this survey can inform adaptations to existing young adult-focused programs or the development of novel interventions to address the increasingly urgent needs of this population.

For individuals and families who are already struggling with high rates of poverty, even small economic changes can have a significant impact on their daily lives and wellbeing. In our study, economic vulnerability appeared to increase during the pandemic with YAHIV reporting lost work and reduced spending on basic needs, mainly on transportation and food. It has been reported that COVID-19 impacted transportation [58] and shopping [59] behaviors as going to markets and shops were associated with a higher risk of contracting COVID-19. In our study, females were more likely to cut down spending on food which may be due to females tending to use more prevention practices [60,61] and/or females often overseeing food shopping for the household [62]. Consequently, there was only a small increase in the number of YAHIV reporting food deprivation since the start of COVID-19. This may be because more than a third of participants had already been experiencing food insecurity prior to COVID-19. Greater increases in food insecurity in the context of COVID-19 were also observed in a study conducted in Kenya and Uganda [63].

Despite broad disruptions to health service delivery globally and in Kenya [64], we found that essential HIV services, particularly access to treatment, were relatively well maintained at the time of the survey. This is likely due to the fact that young adults enrolled in this study were receiving HIV care within a specialized healthcare setting that offered high-quality, personalized services tailored to their age group. In response to the COVID-19 pandemic, healthcare providers implemented exceptional measures to facilitate uninterrupted access to ART, including home delivery, and minimize medication interruptions among these young individuals. Overall, YAHIV in our survey were still able to access their ART medications, though some reported delays in receiving healthcare and stated that COVID-19 and government restrictions made it more difficult to get their medications leading to some missing a dose since the start of COVID-19. Our survey did not explore the exact reasons ART medication was more difficult to obtain, but it may be due to lack of access (transport, money, etc.) and less about the supply of ART medications. This experience stands in contrast to other settings in which clinical care, including HIV services, were negatively impacted thus threatening the progress that has been made to strengthen health systems, most notably HIV care services [65]. Our findings underscore the potential of high-quality medical care tailored to specific groups and the ability of this personalized care system to overcome the challenges of disruptive events like COVID-19.

Prior to the COVID-19 pandemic, adolescent and young adult mental health had emerged as a pressing global concern, with a notable increase in reports of depression and anxiety among young people in both low- and high-income countries [66–69]. Further, YAHIV experience higher rates of mental health disorders compared to uninfected young adults [70–73]. Our survey results indicate that almost a quarter of YAHIV screened positive for potential anxiety and/or depressive disorder warranting further evaluation and follow-up. These rates are similar to those reported by Dyer et al among 20–24-year-olds living with HIV reporting mild-severe depression [41]. While we cannot make any direct associations between these mental health screening results and COVID-19 pandemic, other research indicates that the COVID-19 pandemic and its associated mitigation measures further exacerbated the growing mental health challenges in this population [74–76]. These findings underscore the urgent need to provide mental health services and evaluations for the growing populations of YAHIV [77–79].

One of the major impacts of COVID-19 and the associated restrictions reported in the survey was related to the disruptions and changes in YAHIV living situations. Since the start of COVID-19, a quarter of YAHIV reported living with different people. Concomitantly, there was an increase in verbal arguments or conflicts in their household towards themselves (14%) and others (17%) as well as an increase in physical conflict toward themselves (6%) and others (10%). In Latin American and the Caribbean, 21% of adolescents reported more arguments at home during COVID-19 lockdown [80]. A global systematic review found increases in domestic violence cases during COVID-19 with multiple studies in Africa reporting increases in gender-based violence specifically [81]. Overall, our study showed high rates of violence among both females and males, with higher rates observed among females. In Kenya, high rates of violence among YAHIV, including gender-based violence, have been reported before [82] and during COVID-19 [83]. A study conducted in Kenya, Uganda, Nigeria, and South Africa found that gender-based violence prevention and response services were negatively impacted by COVID-19 and services decreased due to government restrictions [84]. The increases in violence and unsafe settings observed in our study may have negatively impacted the mental health, well-being, and ART adherence [22] of YAHIV who experienced disruptions to their social support networks and services during the COVID-19 restrictions. In our survey, the higher rates of violence reported among females, though not statistically significant, is a notable finding that should be taken into consideration by programs engaging females, including family planning and antenatal care health facilities.

There are limitations to this study. First, the study was conducted at a single healthcare facility in Kisumu, Kenya with a limited sample size, and may not be generalizable to other locations. Furthermore, it was based on a convenience sample of YAHIV actively engaged in care which limits our findings to those who are currently in HIV services. Surveys were self-administered to allow privacy and encourage truthfulness among adolescent respondents. However, YAHIV may have chosen to select socially acceptable answers. Although peer educators were present during the survey administration to provide technology support and answer questions, YAHIV may not have fully understood all of the questions. Additionally, due to funding constraints, this study was limited to only a quantitative survey. While the quantitative analysis provided valuable insights, incorporating a qualitative component would have allowed for a richer understanding of the findings by providing context and depth to the observed results. Future research should consider including qualitative methods, such as interviews or focus groups, to explore YAHIV experiences and perceptions, thereby enriching the findings and supporting a more holistic interpretation.

## Conclusion

Our analysis expands upon the literature exploring the knowledge, attitudes, and practices as well as the impact of COVID-19 among YAHIV in Western Kenya [41,42]. Specifically, our study explored the perceived impact of COVID-19 on young adults who were established in HIV care and recruited directly from an HIV health center. YAHIV in Kenya were knowledgeable about COVID-19 and prevention practices despite inconsistent adherence. Impacts of the pandemic and government restrictions were felt across various aspects of YAHIV’s lives, including disrupted living situations and increased exposure to verbal and physical conflict, including sexual violence. This study provides new insights into the lived experiences of this YAHIV population residing in a low-income setting. Understanding the perceived economic, social and health impacts among this population is essential to recognize what support is needed now and can be offered in the future, especially during other crisis situations. These data can provide insight that can not only inform programming and services for YAHIV including HIV care and treatment, family planning, support group and antenatal services, but also be used as a guide for future pandemic preparedness efforts.

## Supporting information

S1 Checklist (DOCX)

## References

[1] Filip R, Gheorghita Puscaselu R, Anchidin-Norocel L, Dimian M, Savage WK. Global Challenges to Public Health Care Systems during the COVID-19 Pandemic: A Review of Pandemic Measures and Problems. J Pers Med. 2022 Aug 7;12(8):1295. doi: 10.3390/jpm12081295 36013244 PMC9409667

[2] Meinck S, Fraillon J, Strietholt, R. The impact of the COVID-19 pandemic on education: International Evidence from the Responses to Educational Disruption Survey (REDS). UNESCO Publishing; 2022. https://unesdoc.unesco.org/ark:/48223/pf0000380398.

[3] Hosseinzadeh P, Zareipour M, Baljani E, Moradali MR. Social Consequences of the COVID-19 Pandemic. A Systematic Review. Invest Educ Enferm. 2022 Mar;40(1):e10. doi: 10.17533/udea.iee.v40n1e10 35485623 PMC9052715

[4] Duby Z, Bunce B, Fowler C, Bergh K, Jonas K, Dietrich JJ, et al. Intersections between COVID-19 and socio-economic mental health stressors in the lives of South African adolescent girls and young women. Child Adolesc Psychiatry Ment Health. 2022 Mar 26;16(1):23. doi: 10.1186/s13034-022-00457-y 35346316 PMC8959551

[5] Zhu S, Zhuang Y, Ip P. Impacts on Children and Adolescents’ Lifestyle, Social Support and Their Association with Negative Impacts of the COVID-19 Pandemic. Int J Environ Res Public Health. 2021 Apr 29;18(9):4780. doi: 10.3390/ijerph18094780 33947146 PMC8124290

[6] Zhang C, Ye M, Fu Y, Yang M, Luo F, Yuan J, et al. The Psychological Impact of the COVID-19 Pandemic on Teenagers in China. J Adolesc Health. 2020 Dec;67(6):747–755. doi: 10.1016/j.jadohealth.2020.08.026 Epub 2020 Oct 8. 33041204 PMC7543885

[7] Angelina S, Kurniawan A, Agung FH, Halim DA, Wijovi F, Jodhinata C, et al. Adolescents’ mental health status and influential factors amid the Coronavirus Disease pandemic. Clin Epidemiol Glob Health. 2021 Oct-Dec;12:100903. doi: 10.1016/j.cegh.2021.100903 Epub 2021 Nov 12. 34786520 PMC8588605

[8] Porter C, Favara M, Hittmeyer A, Scott D, Sánchez Jiménez A, Ellanki R, et al. Impact of the COVID-19 pandemic on anxiety and depression symptoms of young people in the global south: evidence from a four-country cohort study. BMJ Open. 2021 Apr 15;11(4):e049653. doi: 10.1136/bmjopen-2021-049653 33858874 PMC8053815

[9] Banati P, Jones N, Youssef S. Intersecting Vulnerabilities: The Impacts of COVID-19 on the Psycho-emotional Lives of Young People in Low- and Middle-Income Countries. Eur J Dev Res. 2020;32(5):1613–1638. doi: 10.1057/s41287-020-00325-5 Epub 2020 Nov 9. 33191985 PMC7649704

[10] Kakaei H, Nourmoradi H, Bakhtiyari S, Jalilian M, Mirzaei A. Effect of COVID-19 on food security, hunger, and food crisis. COVID-19 and the Sustainable Development Goals. 2022:3–29. Epub 2022 Jul 29.

[11] The World Bank. COVID-19 to Add as Many as 150 Million Extreme Poor by 2021. Press Release. 7 October 2020. https://www.worldbank.org/en/news/press-release/2020/10/07/covid-19-to-add-as-many-as-150-million-extreme-poor-by-2021.

[12] UNICEF. COVID-19 and School Closures: One year of education disruption. Report. 2 March 2021. https://data.unicef.org/resources/one-year-of-covid-19-and-school-closures/.

[13] Wang D, Adedokun OA, Millogo O, Madzorera I, Hemler EC, Workneh F, et al. The Continued Impacts of the COVID-19 Pandemic on Education and Mental Health Among Sub-Saharan African Adolescents. J Adolesc Health. 2023 Apr;72(4):535–543. doi: 10.1016/j.jadohealth.2022.11.012 Epub 2022 Nov 28. 36635187 PMC9701646

[14] UNICEF. COVID-19: Are children able to continue learning during school closures? A global analysis of the potential reach of remote learning policies. Report. 26 August 2020. https://data.unicef.org/resources/remote-learning-reachability-factsheet/.

[15] Ahinkorah BO, Hagan JE Jr, Ameyaw EK, Seidu AA, Schack T. COVID-19 Pandemic Worsening Gender Inequalities for Women and Girls in Sub-Saharan Africa. Front Glob Womens Health. 2021 Jul 29;2:686984. doi: 10.3389/fgwh.2021.686984 34816232 PMC8594039

[16] Abdalla S, Katz EG, Hartley A, Darmstadt GL. Gender and the impact of COVID-19 on demand for and access to health care: Intersectional analysis of before-and-after data from Kenya, Nigeria, and South Africa. J Glob Health. 2022 Aug 13;12:05024. doi: 10.7189/jogh.12.05024 35959957 PMC9373834

[17] Alvi MF, Gupta S, Barooah P, Ringler C, Bryan E, Meinzen D, et al. 2022. Gendered impacts of COVID-19: Insights from 7 countries in Sub-Saharan Africa and South Asia. Washington, DC: International Food Policy Research Institute (IFPRI). 10.2499/p15738coll2.135042

[18] Western Cape Department of Health in collaboration with the National Institute for Communicable Diseases, South Africa. Risk Factors for Coronavirus Disease 2019 (COVID-19) Death in a Population Cohort Study from the Western Cape Province, South Africa. Clin Infect Dis. 2021 Oct 5;73(7):e2005–e2015. Erratum in: Clin Infect Dis. 2022 Apr 9;74(7):1321. doi: 10.1093/cid/ciaa1198 32860699 PMC7499501

[19] Spinelli MA, Jones BLH, Gandhi M. COVID-19 Outcomes and Risk Factors Among People Living with HIV. Curr HIV/AIDS Rep. 2022 Oct;19(5):425–432. doi: 10.1007/s11904-022-00618-w Epub 2022 Aug 5. 35930187 PMC9362624

[20] Toska E, Gittings L, Hodes R, Cluver LD, Govender K, Chademana KE, et al. Resourcing resilience: social protection for HIV prevention amongst children and adolescents in Eastern and Southern Africa. Afr J AIDS Res. 2016 Jul;15(2):123–40. doi: 10.2989/16085906.2016.1194299 Erratum for: Afr J AIDS Res. 2016 Sep;15(3):314. Erratum in: Afr J AIDS Res. 2016 Sep;15(3):314. 27399042 PMC5558245

[21] Bermudez LG, Jennings L, Ssewamala FM, Nabunya P, Mellins C, McKay M. Equity in adherence to antiretroviral therapy among economically vulnerable adolescents living with HIV in Uganda. AIDS Care. 2016 Mar;28 Suppl 2(sup2):83–91. doi: 10.1080/09540121.2016.1176681 27392003 PMC4940111

[22] Cluver L, Shenderovich Y, Toska E, Rudgard WE, Zhou S, Orkin M, et al. Clinic and care: associations with adolescent antiretroviral therapy adherence in a prospective cohort in South Africa. AIDS. 2021 Jul 1;35(8):1263–1271. doi: 10.1097/QAD.0000000000002882 33730747 PMC8183481

[23] Kang E, Mellins CA, Kim W, Dolezal C, Kindler C, Leu CS, et al. Navigating Stigma Trajectory and Mental Health Among Young Adults Living with Perinatal HIV in New York City. AIDS Behav. 2021 Nov;25(11):3712–3720. doi: 10.1007/s10461-021-03166-3 Epub 2021 Feb 1. 33523346 PMC8638371

[24] Trief PM, Kalichman SC, Wang D, et al. Medication adherence in young adults with youth-onset type 2 diabetes: iCount, an observational study. Diabetes Res Clin Pract. 2022;184:109216. doi: 10.1016/j.diabres.2022.109216 35085644

[25] Ferrand RA, Briggs D, Ferguson J, Penazzato M, Armstrong A, MacPherson P, et al. Viral suppression in adolescents on antiretroviral treatment: review of the literature and critical appraisal of methodological challenges. Trop Med Int Health. 2016 Mar;21(3):325–33. doi: 10.1111/tmi.12656 Epub 2016 Jan 10. 26681359 PMC4776345

[26] Zanoni BC, Archary M, Buchan S, Katz IT, Haberer JE. Systematic review and meta-analysis of the adolescent HIV continuum of care in South Africa: the Cresting Wave. BMJ Glob Health. 2016 Oct 24;1(3):e000004. doi: 10.1136/bmjgh-2015-000004 28588949 PMC5321340

[27] Reif LK, Abrams EJ, Arpadi S, Elul B, McNairy ML, Fitzgerald DW, et al. Interventions to Improve Antiretroviral Therapy Adherence Among Adolescents and Youth in Low- and Middle-Income Countries: A Systematic Review 2015–2019. AIDS Behav. 2020 Oct;24(10):2797–2810. doi: 10.1007/s10461-020-02822-4 32152815 PMC7223708

[28] Griffiths K, Ford N. Provision of antiretroviral care to displaced populations in humanitarian settings: a systematic review. Med Confl Surviv. 2013 Jul-Sep;29(3):198–215. doi: 10.1080/13623699.2013.813108 . 24133930

[29] Auld AF, Valerie P, Robin EG, Shiraishi RW, Dee J, Antoine M, et al. Retention Throughout the HIV Care and Treatment Cascade: From Diagnosis to Antiretroviral Treatment of Adults and Children Living with HIV-Haiti, 1985–2015. Am J Trop Med Hyg. 2017 Oct;97(4_Suppl):57–70. doi: 10.4269/ajtmh.17-0116 29064357 PMC5676635

[30] Anthonj C, Nkongolo OT, Schmitz P, Hango JN, Kistemann T. The impact of flooding on people living with HIV: a case study from the Ohangwena Region, Namibia. Glob Health Action. 2015 Mar 25;8:26441. doi: 10.3402/gha.v8.26441 25813771 PMC4375215

[31] Orievulu KS, Ayeb-Karlsson S, Ngema S, Baisley K, Tanser F, Ngwenya N, et al. Exploring linkages between drought and HIV treatment adherence in Africa: a systematic review. Lancet Planet Health. 2022 Apr;6(4):e359–e370. doi: 10.1016/S2542-5196(22)00016-X 35397224 PMC7612934

[32] International Monetary Fund. Policy Responses to COVID-19. 2021. https://www.imf.org/en/Topics/imf-and-covid19/Policy-Responses-to-COVID-19.

[33] Bloomberg News. Kenya Reports First Case of Coronavirus. 13 March 2020. https://www.bloomberg.com/news/articles/2020-03-13/kenyan-health-secretary-reports-first-case-of-coronavirus#xj4y7vzkg.

[34] Aluga MA. Coronavirus Disease 2019 (COVID-19) in Kenya: Preparedness, response and transmissibility. J Microbiol Immunol Infect. 2020 Oct;53(5):671–673. doi: 10.1016/j.jmii.2020.04.011 Epub 2020 Apr 20. 32331980 PMC7167550

[35] Kenya Ministry of Health. National AIDS & STI Control Program. COVID‐19 guidance on comprehensive HIV service delivery. 24 March 2020. https://www.differentiatedservicedelivery.org/resources/covid-19-dsd-resources-national-guidance/.

[36] Center for Systems Science and Engineering (CSSE) at Johns Hopkins University (JHU). COVID-19 Dashboard. https://www.arcgis.com/apps/dashboards/bda7594740fd40299423467b48e9ecf6. 10.1016/S1473-3099(22)00434-0 PMC9432867 36057267

[37] Coronavirus Resource Center at Johns Hopkins University (JHU). Kenya Overview. https://coronavirus.jhu.edu/region/kenya.

[38] Mwakisha J, Adika B, Nyawade S, Phori PM, Nidjergou NN, Silouakadila C, et al. Kenya’s Experience: Factors Enabling and Impeding the COVID-19 Response. Health Promot Pract. 2023 Feb 3:15248399221117566. doi: 10.1177/15248399221117566 Epub ahead of print. 36734323 PMC9899671

[39] Karijo E, Wamugi S, Lemanyishoe S, Njuki J, Boit F, Kibui V, et al. Knowledge, attitudes, practices, and the effects of COVID-19 among the youth in Kenya. BMC Public Health. 2021 May 30;21(1):1020. doi: 10.1186/s12889-021-11067-2 34053442 PMC8164891

[40] Decker MR, Wood SN, Thiongo M, Byrne ME, Devoto B, Morgan R, et al. Gendered health, economic, social and safety impact of COVID-19 on adolescents and young adults in Nairobi, Kenya. PLoS One. 2021 Nov 9;16(11):e0259583. doi: 10.1371/journal.pone.0259583 34752473 PMC8577767

[41] Dyer J, Wilson K, Badia J, Agot K, Neary J, Njuguna I, et al. The Psychosocial Effects of the COVID-19 Pandemic on Youth Living with HIV in Western Kenya. AIDS Behav. 2021 Jan;25(1):68–72. doi: 10.1007/s10461-020-03005-x 32816193 PMC7438976

[42] Enane LA, Apondi E, Aluoch J, Bakoyannis G, Lewis Kulzer J, Kwena Z, et al. Social, economic, and health effects of the COVID-19 pandemic on adolescents retained in or recently disengaged from HIV care in Kenya. PLoS One. 2021 Sep 10;16(9):e0257210. doi: 10.1371/journal.pone.0257210 34506555 PMC8432853

[43] AIDSinfo team. UNAIDS Estimates. 2022. https://aidsinfo.unaids.org/?did=5f010acb629b296603d55aa6&r=world&t=2021&tb=d&bt=dnli&ts=0,0&tr=world&aid=5f010bcf629b296603d55aa9&sav=Population.

[44] National AIDS and STI Control Programme (NASCOP). Kenya Population-based HIV Impact Assessment (KENPHIA) 2018: Final Report. Nairobi: NASCOP; August 2022. https://phia.icap.columbia.edu/kenya-final-report-2018/.

[45] National Council for Population and Development. Increasing New HIV Infections among Young People: A Threat to the Realization of Demographic Dividend in Kenya. Policy Brief. June 2018. https://ncpd.go.ke/wp-content/uploads/2021/02/59-PB-Increasing-new-HIV-infections-among.pdf.

[46] Wilson K, Onyango A, Mugo C, Guthrie B, Slyker J, Richardson B, et al. Kenyan HIV Clinics With Youth-Friendly Services and Trained Providers Have a Higher Prevalence of Viral Suppression Among Adolescents and Young Adults: Results From an Observational Study. J Assoc Nurses AIDS Care. 2022 Jan-Feb 01;33(1):45–53. doi: 10.1097/JNC.0000000000000302 34939987 PMC10329499

[47] U.S. Department of State. PEPFAR. Operation Triple Zero: Empowering Adolescents and Young People Living with HIV to Take Control of Their Health. 16 November 2018. https://www.state.gov/operation-triple-zero-empowering-adolescents-and-young-people-living-with-hiv-to-take-control-of-their-health-in-kenya/.

[48] World Health Organization. Adolescent-friendly health services for adolescents living with HIV: from theory to practice. Technical Brief. 27 November 2019. https://www.who.int/publications/i/item/adolescent-friendly-health-services-for-adolescents-living-with-hiv.

[49] World Health Organization. Making health services adolescent friendly: Developing national quality standards for adolescent friendly health services. Guidebook. https://www.who.int/publications/i/item/9789241503594.

[50] World Health Organization. Survey tool and guidance: rapid, simple, flexible behavioural insights on COVID-19. 29 July 2020. https://www.who.int/europe/tools-and-toolkits/who-tool-for-behavioural-insights-on-covid-19.

[51] Fields JF, Hunter-Childs J, Tersine A, Sisson J, Parker E, Velkoff V, et al. Design and Operation of the 2020 Household Pulse Survey, 2020. U.S. Census Bureau. Preliminary Draft. 11 March 2020. https://www2.census.gov/programs-surveys/demo/technical-documentation/hhp/2020_HPS_Background.pdf.

[52] National HIV Curriculum. Generalized Anxiety Disorder 2-item (GAD-2). 2023. https://www.hiv.uw.edu/page/mental-health-screening/gad-2.

[53] Kroenke K, Spitzer RL, Williams JB. The Patient Health Questionnaire-2: Validity of a Two-Item Depression Screener. Medical Care. 2003;41:1284–92. doi: 10.1097/01.MLR.0000093487.78664.3C 14583691

[54] World Health Organization. Impact of COVID-19 on people’s livelihoods, their health and our food systems. 13 October 2020. https://www.who.int/news/item/13-10-2020-impact-of-covid-19-on-people’s-livelihoods-their-health-and-our-food-systems.

[55] World Health Organization. The impact of COVID-19 on global health goals. 20 May 2021. https://www.who.int/news-room/spotlight/the-impact-of-covid-19-on-global-health-goals.

[56] Kaye AD, Okeagu CN, Pham AD, Silva RA, Hurley JJ, Arron BL, et al. Economic impact of COVID-19 pandemic on healthcare facilities and systems: International perspectives. Best Pract Res Clin Anaesthesiol. 2021 Oct;35(3):293–306. doi: 10.1016/j.bpa.2020.11.009 Epub 2020 Nov 17. 34511220 PMC7670225

[57] Moynihan R, Sanders S, Michaleff ZA, Scott AM, Clark J, To EJ, et al. Impact of COVID-19 pandemic on utilisation of healthcare services: a systematic review. BMJ Open. 2021 Mar 16;11(3):e045343. doi: 10.1136/bmjopen-2020-045343 33727273 PMC7969768

[58] Elbany M, Elhenawy Y. Analyzing the ultimate impact of COVID-19 in Africa. Case Stud Transp Policy. 2021 Jun;9(2):796–804. doi: 10.1016/j.cstp.2021.03.016 Epub 2021 Apr 6. 33842205 PMC8021502

[59] Ben Hassen T, El Bilali H, Allahyari MS, Kamel IM, Ben Ismail H, Debbabi H, et al. Gendered Impacts of the COVID-19 Pandemic on Food Behaviors in North Africa: Cases of Egypt, Morocco, and Tunisia. Int J Environ Res Public Health. 2022 Feb 15;19(4):2192. doi: 10.3390/ijerph19042192 35206378 PMC8872065

[60] Galasso V, Pons V, Profeta P, Becher M, Brouard S, Foucault M. Gender differences in COVID-19 attitudes and behavior: Panel evidence from eight countries. Proc Natl Acad Sci U S A. 2020 Nov 3;117(44):27285–27291. doi: 10.1073/pnas.2012520117 Epub 2020 Oct 15. 33060298 PMC7959517

[61] Guenther B, Galizzi MM, Sanders JG. Heterogeneity in Risk-Taking During the COVID-19 Pandemic: Evidence From the UK Lockdown. Front Psychol. 2021 Apr 1;12:643653. doi: 10.3389/fpsyg.2021.643653 33868115 PMC8046913

[62] Bukachi SA, Ngutu M, Muthiru AW, Lépine A, Kadiyala S, Domínguez-Salas P. Gender and sociocultural factors in animal source foods (ASFs) access and consumption in lower-income households in urban informal settings of Nairobi, Kenya. J Health Popul Nutr. 2022 Jul 11;41(1):30. doi: 10.1186/s41043-022-00307-9 35818082 PMC9275060

[63] Kansiime MK, Tambo JA, Mugambi I, Bundi M, Kara A, Owuor C. COVID-19 implications on household income and food security in Kenya and Uganda: Findings from a rapid assessment. World Dev. 2021 Jan;137:105199. doi: 10.1016/j.worlddev.2020.105199 Epub 2020 Sep 18. 32982018 PMC7500897

[64] Barasa E, Kazungu J, Orangi S, Kabia E, Ogero M, Kasera K. Indirect health effects of the COVID-19 pandemic in Kenya: a mixed methods assessment. BMC Health Serv Res. 2021 Jul 26;21(1):740. doi: 10.1186/s12913-021-06726-4 34311716 PMC8311400

[65] Ojukwu E, Pashaei A, Maia JC, Omobhude OF, Tawfik A, Nguyen Y. Repercussions of the COVID-19 pandemic on the HIV care continuum and related factors in economically disadvantaged nations: an integrated analysis using mixed-methods systematic review. Eur J Med Res. 2024 Jun 26;29(1):346. doi: 10.1186/s40001-024-01917-1 38926792 PMC11202375

[66] Gunnell D, Kidger J, Elvidge H. Adolescent mental health in crisis. BMJ. 2018 Jun 19;361:k2608. doi: 10.1136/bmj.k2608 . 29921659

[67] McGorry PD, Mei C. Tackling the youth mental health crisis across adolescence and young adulthood. BMJ. 2018 Sep 6;362:k3704. doi: 10.1136/bmj.k3704 . 30190368

[68] UNICEF. Ensuring mental health and well-being in an adolescent’s formative years can foster a better transition from childhood to adulthood. Report. October 2021. https://data.unicef.org/topic/child-health/mental-health/.

[69] Patel V, Saxena S, Lund C, Thornicroft G, Baingana F, Bolton P, et al. The Lancet Commission on global mental health and sustainable development. The Lancet, 387(10024), 1143–1145. 10.1016/S0140-6736(16)00208-7 27025320

[70] Kamau JW, Kuria W, Mathai M, Atwoli L, Kangethe R. Psychiatric morbidity among HIV-infected children and adolescents in a resource-poor Kenyan urban community. AIDS Care. 2012;24(7):836–42. doi: 10.1080/09540121.2011.644234 Epub 2012 Jan 31. . 22292795

[71] Gross IM, Hosek S, Richards MH, Fernandez MI. Predictors and Profiles of Antiretroviral Therapy Adherence Among African American Adolescents and Young Adult Males Living with HIV. AIDS Patient Care STDS. 2016 Jul;30(7):324–38. doi: 10.1089/apc.2015.0351 27410496 PMC4948258

[72] UNICEF, University of Cape Town, University of Oxford. Mental health and antiretroviral treatment adherence among adolescents living with HIV. Policy Brief. 2021. https://www.unicef.org/esa/media/10231/file/Mental-Health-Treatment-Adhrence-Policy-Brief-2021.pdf.

[73] Vreeman RC, McCoy BM, Lee S. Mental health challenges among adolescents living with HIV. J Int AIDS Soc. 2017 May 16;20(Suppl 3):21497. doi: 10.7448/IAS.20.4.21497 28530045 PMC5577712

[74] Duby Z, Bunce B, Fowler C, Bergh K, Jonas K, Dietrich JJ, et al. Intersections between COVID-19 and socio-economic mental health stressors in the lives of South African adolescent girls and young women. Child Adolesc Psychiatry Ment Health 16, 23 (2022). doi: 10.1186/s13034-022-00457-y 35346316 PMC8959551

[75] Zuilkowski SS, Quinones S, Kihanzah H, Marwerwe G, Prencipe L, Kajula L, et al. Economic vulnerabilities, mental health, and coping strategies among Tanzanian youth during COVID-19. BMC Public Health. 2024 Feb 22;24(1):577. doi: 10.1186/s12889-024-18074-z 38388862 PMC10885560

[76] Meherali S, Punjani N, Louie-Poon S, Abdul Rahim K, Das JK, Salam RA, et al. Mental Health of Children and Adolescents Amidst COVID-19 and Past Pandemics: A Rapid Systematic Review. Int J Environ Res Public Health. 2021 Mar 26;18(7):3432. doi: 10.3390/ijerph18073432 33810225 PMC8038056

[77] Harrison L, Carducci B, Klein JD, Bhutta ZA. Indirect effects of COVID-19 on child and adolescent mental health: an overview of systematic reviews. BMJ Glob Health. 2022 Dec;7(12):e010713. doi: 10.1136/bmjgh-2022-010713 36585030 PMC9808753

[78] Jones EAK, Mitra AK, Bhuiyan AR. Impact of COVID-19 on Mental Health in Adolescents: A Systematic Review. Int J Environ Res Public Health. 2021 Mar 3;18(5):2470. doi: 10.3390/ijerph18052470 33802278 PMC7967607

[79] Tang S, Xiang M, Cheung T, Xiang YT. Mental health and its correlates among children and adolescents during COVID-19 school closure: The importance of parent-child discussion. J Affect Disord. 2021 Jan 15;279:353–360. doi: 10.1016/j.jad.2020.10.016 Epub 2020 Oct 12. 33099049 PMC7550131

[80] UNICEF LACRO. Youth speak up about violence during COVID-19. https://www.unicef.org/lac/en/youth-speak-about-violence-during-covid-19.

[81] Kourti A, Stavridou A, Panagouli E, Psaltopoulou T, Spiliopoulou C, Tsolia M, et al. Domestic Violence During the COVID-19 Pandemic: A Systematic Review. Trauma Violence Abuse. 2023 Apr;24(2):719–745. doi: 10.1177/15248380211038690 Epub 2021 Aug 17. 34402325 PMC10011925

[82] Annor FB, Chiang LF, Oluoch PR, Ma’g’oli V, Mogaka M, Mwangi M, et al. Changes in prevalence of violence and risk factors for violence and HIV among children and young people in Kenya: a comparison of the 2010 and 2019 Kenya Violence Against Children and Youth Surveys. Lancet Glob Health. 2022 Jan;10(1):e124–e133. doi: 10.1016/S2214-109X(21)00457-5 Epub 2021 Nov 22. 34822755 PMC9791664

[83] Decker MR, Bevilacqua K, Wood SN, Ngare GW, Thiongo M, Byrne ME, et al. Gender-based violence during COVID-19 among adolescent girls and young women in Nairobi, Kenya: a mixed-methods prospective study over 18 months. BMJ Glob Health. 2022 Feb;7(2):e007807. doi: 10.1136/bmjgh-2021-007807 35210310 PMC8882641

[84] Roy CM, Bukuluki P, Casey SE, Jagun MO, John NA, Mabhena N, et al. Impact of COVID-19 on Gender-Based Violence Prevention and Response Services in Kenya, Uganda, Nigeria, and South Africa: A Cross-Sectional Survey. Front Glob Womens Health. 2022 Jan 27;2:780771. doi: 10.3389/fgwh.2021.780771 35156086 PMC8829509

